# Dexamethasone inhibits IL-8 via glycolysis and mitochondria-related pathway to regulate inflammatory pain

**DOI:** 10.1186/s12871-023-02277-9

**Published:** 2023-09-18

**Authors:** Ren He, Xiaohan Li, Silun Zhang, Yuqiang Liu, Qingsheng Xue, Yan Luo, Buwei Yu, Xiongjuan Li, Zhiheng Liu

**Affiliations:** 1 Department of Anesthesiology, Shenzhen Second People’s Hospital, The First Affiliated Hospital of Shenzhen University, Health Science Center, No. 3002, Futian District, Shenzhen, Guangdong Province 518035 China; 2 Department of Anesthesiology, Ruijin Hospital Affiliated to Shanghai Jiaotong University, Shanghai, 200025 China

**Keywords:** Dexamethasone, Mitochondrial pathway, Glycolysis, IL-8, Inflammatory pain

## Abstract

**Background:**

Dexamethasone (Dexa) has been recently found to exert an analgesic effect, whose action is closely related to IL-8. However, whether dexamethasone induces antinociception via glycolysis and mitochondria-related pathways is still unclear.

**Methods:**

Right hind paw inflammatory pain in mice was induced by intraplantar injection of Freund’s Complete Adjuvant (FCA). Von Frey test was then used to measure the paw withdrawal threshold. The detection of glycolysis and mitochondrial pathway-related proteins and IL-8 were determined by Western blot and ELISA. The potential interaction between Dexa and fructose-1,6-bisphosphate (FBP, a PKM2 activator) was examined by simulation predictions using molecular docking.

**Results:**

Intrathecal administration of Dexa (20 µg/20 µL) had an obvious analgesic effect in FCA-treated mice, which was counteracted by the glycolysis inhibitor 2-deoxyglucose (2-DG, 5 mg/20 µL) or the mitochondria-related pathway inhibitor oligomycin complex (Oligo, 5 µg/20 µL). In the glycolysis pathway, Dexa decreased GLUT3 and had no impact on HIF-1α expression during FCA-induced inflammation. Additionally, Dexa further increased the PKM2 level, accompanied by the formation of hydrogen bonds between Dexa and the PKM2 activator fructose-1,6-bisphosphate (FBP). In the mitochondrial pathway, Dexa downregulated the expression of Mfn2 protein but not the PGC-1α and SIRT-1 levels in the spinal cord. Moreover, both 2-DG and Oligo decreased Mfn2 expression. Finally, IL-8 level was reduced by the single or combined administration of Dexa, 2-DG, and Oligo.

**Conclusion:**

Dexa attenuated IL-8 expression via glycolysis and mitochondrial pathway-related proteins, thus mediating the analgesic effect during inflammatory pain.

**Supplementary Information:**

The online version contains supplementary material available at 10.1186/s12871-023-02277-9.

## Introduction

Dexamethasone (Dexa) is a glucocorticoid hormone commonly used clinically for its anti-inflammatory, anti-allergic, anti-shock, and immunosuppression effects. It was also found to have intrathecal analgesic effect recently, which can be safely used in local nerve block [1, 2]. Its application is of significant value as local drug delivery is frequently preferred over systemic administration [3]. However, the effect of dexamethasone on glycolysis and the mitochondria-related pathway during inflammatory pain have not been studied. Thus, in the present study, we aimed to address this knowledge gap.

Energy metabolism is essential for normal cell functioning. Glucose metabolism serves fundamental roles in intracellular energy production. Glycolysis pathway, which includes anaerobic and aerobic catabolism [4], is involved in glucose metabolism and immune response [5]. As a glucose sensor, glucose transporter isoform 3 (GLUT3) is ubiquitously expressed in central nervous system and predominantly localized in neurons, mediating glucose transport with the highest affinity for glucose and pain sensation [6]. Pyruvate kinase M2 (PKM2) is a key isoform of the terminal glycolytic enzyme that could also be considered to play an important role in pain initiation or modulation. Previous studies have demonstrated that PKM2 can serve as the new therapeutic target or biomarker for rheumatoid arthritis [7]. Hypoxia inducible factor-1α (HIF-1α) is able to regulate the expression of glycolytic enzymes and the metabolic shift of mitochondrial respiration towards glycolysis [8]. It has been reported that HIF-1α is able to mitigate diabetic neuropathic pain [9].

Mitochondrial (the intrinsic) pathway is also important for cellular energy metabolism, the apoptotic pathway, and inflammatory responses [10]. Mitofusin 2 (Mfn2) is considered to be a significant regulator of cellular metabolism and inflammation, and the downregulation of Mfn2 can reduce inflammation in osteoarthritis [11]. However, its role in inflammatory pain remains poorly understood. Previous studies found that the peroxisome proliferators-activated receptor γ coactivator alpha (PGC-1α) and the silent mating-type information regulation 2 homolog-1 (SIRT1) are also involved in the control of mitochondrial biogenesis, thus regulating peripheral neuropathy and inflammatory pain [12, 13].

As a pro-inflammatory cytokine, interleukin 8 (IL-8) might play a significant role in inflammatory pathogenesis, which leads to inflammatory pain [14]. Dexa was established to reduce the IL-8 level under inflammatory conditions [15]. However, whether Dexa affect IL-8 levels via the glycolytic or mitochondrial pathway in inflammation-induced pain has remained unknown. Therefore, here, we aimed to elucidate whether Dexa exert effects on IL-8 via energy metabolism-related pathways.

## Methods

### Animals

This experiment was approved by the local animal ethics committee (number 20,210,006). Before the experiment, a total number of 80 adult male healthy C57/BL6J mice (6–8-week-old, weighing 20–30 g) were allowed to adjust to the test environment for one week under standard conditions (12-h light/dark cycle, temperature of 22 ℃ ± 2 ℃, and humidity of 55% ± 5%). All mice were given *ad libitum* access to feed and water.

### Induction of inflammatory pain

Right hind paw inflammation was induced by Freund’s complete adjuvant (FCA, Sigma, USA) containing 1 mg/mL heat-killed *Mycobacterium tuberculosis*. The mice received a right intraplantar injection of 20 uL of FCA under isoflurane anesthesia (1–3%, Baxter) delivered with oxygen at a flow rate of 1 L/min. The left hind paw remained intact for self-control.

### Reagents and experimental protocols

Dexamethasone (Dexa, glucocorticoid receptor agonist) solution was obtained from Rongsheng Pharmaceutical Co., Ltd (Jiaozuo, China); 2-deoxyglucose (2-DG, glycolysis inhibitor) and oligomycin complex (Oligo, mitochondrial pathway inhibitor) were purchased from APExBIO (Houston, USA). Then, 2-DG and Oligo were dissolved in saline and 1% ethanol with saline, respectively. Mice were randomly assigned to seven groups: control (no treatment), FCA, Dexa (20 µg/20 µL), 2-DG (5 mg/20 µL), Dexa + 2-DG (20 µg + 5 mg/20 µL), Oligo (5 µg/20 µL), and Dexa + Oligo (20 µg + 5 µg/20 µL). The aforementioned compounds were administered intrathecally *via* the L5-6 lumbar interspace identified by the tail flick reflex under isoflurane inhalation anesthesia four days post-FCA treatment. Further, we examined the impact of pain using the von Frey filament test. Subsequently, all the mice were sacrificed five days post-FCA injection, and the lumbar spinal cords (L3 ~ 5) were obtained to investigate the expression of glycolytic and mitochondrial pathway-related proteins, as well as that of the downstream cytokine IL-8.

### Von Frey filament tests

The paw withdrawal mechanical threshold (PWMT) was measured using the von Frey filament test (Stoelting, IL, USA). Briefly, the mice were placed and habituated to a wire mesh floor with a square plastic box. Their pain behavior was tested using the up-and-down method with the following resulting pattern of positive of negative modes: X = withdrawal, O = no withdrawal. The 50% response threshold was interpolated using the following formula: 50% g threshold = (10(sf + kδ))/10,000.

### Western blot

The mice were euthanized with overdose isoflurane anesthesia, and the L3-5 lumbar spinal cords were obtained. Then, the concentration of the homogenized protein extract was determined using a BCA assay kit (Beyotime, Shanghai, China). Equal protein quantities were denatured, separated by SDS-PAGE electrophoresis, and electrotransferred to membranes. These membranes were blocked with 5% nonfat milk in TBST for 2 h at room temperature and probed with rabbit anti-GLUT3, anti-PKM2, anti-GAPDH, anti-β-actin, and mouse anti-HIF-1α antibodies (1:1000, Affinity, USA). Subsequently, the blots were incubated with secondary HRP goat anti-rabbit, goat anti-mouse IgG (1:10000, Affinity) in 5% nonfat milk/TBST at room temperature for 1 h. Finally, the blots were visualized with ECL reagent (Millipore, USA) using a chemiluminometric detector (Tianneng, Shanghai, China). Quantitative image analysis was performed with Image J (National Institutes of Health, Bethesda, MD, USA).

### ELISA

The concentration levels of spinal Mfn2, PGC-1α, SIRT1, and IL-8 were determined by enzyme-linked immunosorbent assay (ELISA) using mouse an Mfn2 ELISA kit (ELK Biotechnology, Wuhan, China), mouse PGC-1α ELISA kit (ELK Biotechnology, Wuhan, China), mouse SIRT1 ELISA kit (Elabscience, USA), and mouse IL-8 ELISA kit (Meimian, Jiangsu, China), following the instructions of the manufacturers.

### Molecular docking

Molecular docking between Dexa and fructose-1,6-bisphosphate (FBP, PKM2 activator) were performed using the Autodock 4.2 package. The 2D structure of Dexa (CID:5743) was obtained from the PubChem database and then converted into 3D *mol format using the Open Babel graphical user interface software. The 3D structure of FBP (PDB ID:5et5) was downloaded from the RCSB Protein Data Bank in *PDB format. The operation procedures for molecular docking were performed as described previously [16]. The output data were rendered by PyMOL, and the 2D image of Dexa-FBP interactions were visualized though Discovery Studio software.

### Statistical analysis

All data were expressed using mean ± standard deviation (SD). Differences in Von Frey behavior data were analyzed using repeat measures ANOVA. For other comparisons between multiple groups, data were performed using ANOVA test. Statistical analyses were performed using Sigma Stat software (SPSS, Germany). And the graphs were drawn using the GraphPad Prism 7.0 software (GraphPad Software Inc., CA, USA). *P*<0.05 was considered to indicate a statistically significant difference.

## Results

### Analgesia effect of Dexa was counteracted by a glycolysis or mitochondrial pathway inhibitor

**Fig. 1:**
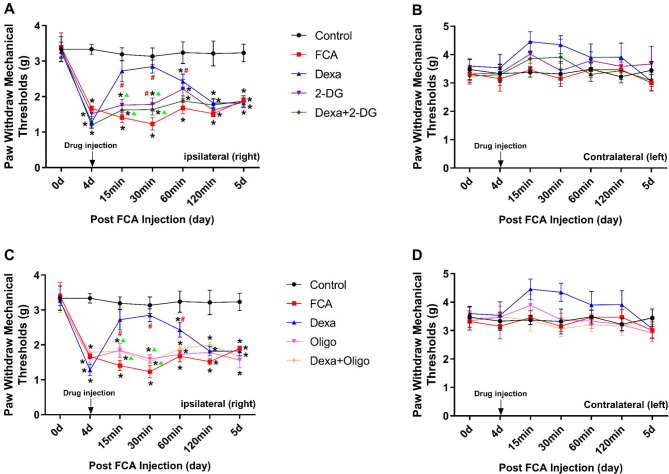
Intrathecal injection of Dexa alleviated FCA-induced inflammatory pain. (**A–D**) The effects of Dexa (20 µg/20 µL), 2-deoxyglucose (2-DG, 5 mg/20 µL, glycolysis inhibitor) or oligomycin complex (Oligo, 5 µg/20 µL, mitochondrial pathway inhibitor) following their single or combined administration on ipsilateral and contralateral paw withdrawal mechanical thresholds (PWMTs). Dexa significantly decreased the pain in the ipsilateral hind paw of the FCA-treated mice, which was reversed by 2-DG or Oligo administration (**A, C**), whereas the contralateral side remained unchanged (**B, D**). * *P*<0.05 versus control; # *P*<0.05 versus FCA group; and ▲ *P*<0.05 versus Dexa group in repeated measures ANOVA. Data are presented as mean ± SD (n = 6). FCA, Freund’s complete adjuvant

In ipsilateral hind paw, there were significant reductions in PWMT with FCA-treated mice versus controls. The intrathecal injection of Dexa (20 µg/20 µL) exerted an obvious analgesic effect 60 min after drug administration as compared to FCA group. The glycolysis inhibitor (2-DG, 5 mg/20 µL) displayed antinociceptive effect in 30 min, and the mitochondrial pathway inhibitor (Oligo, 5 µg/20 µL) alone did not reverse the reduced PWMT in the FCA-treated mice. The drug combination (Dexa + 2-DG or Dexa + Oligo) attenuated the Dexa-induced antinociception (Fig. 1A, C). In addition, Dexa exerted no effect on the PWMT in the contralateral hind paw (Fig. 1B, D).

### Dexa induces changes in the glycolysis pathway-related proteins of GLUT3 and PKM2

**Fig. 2:**
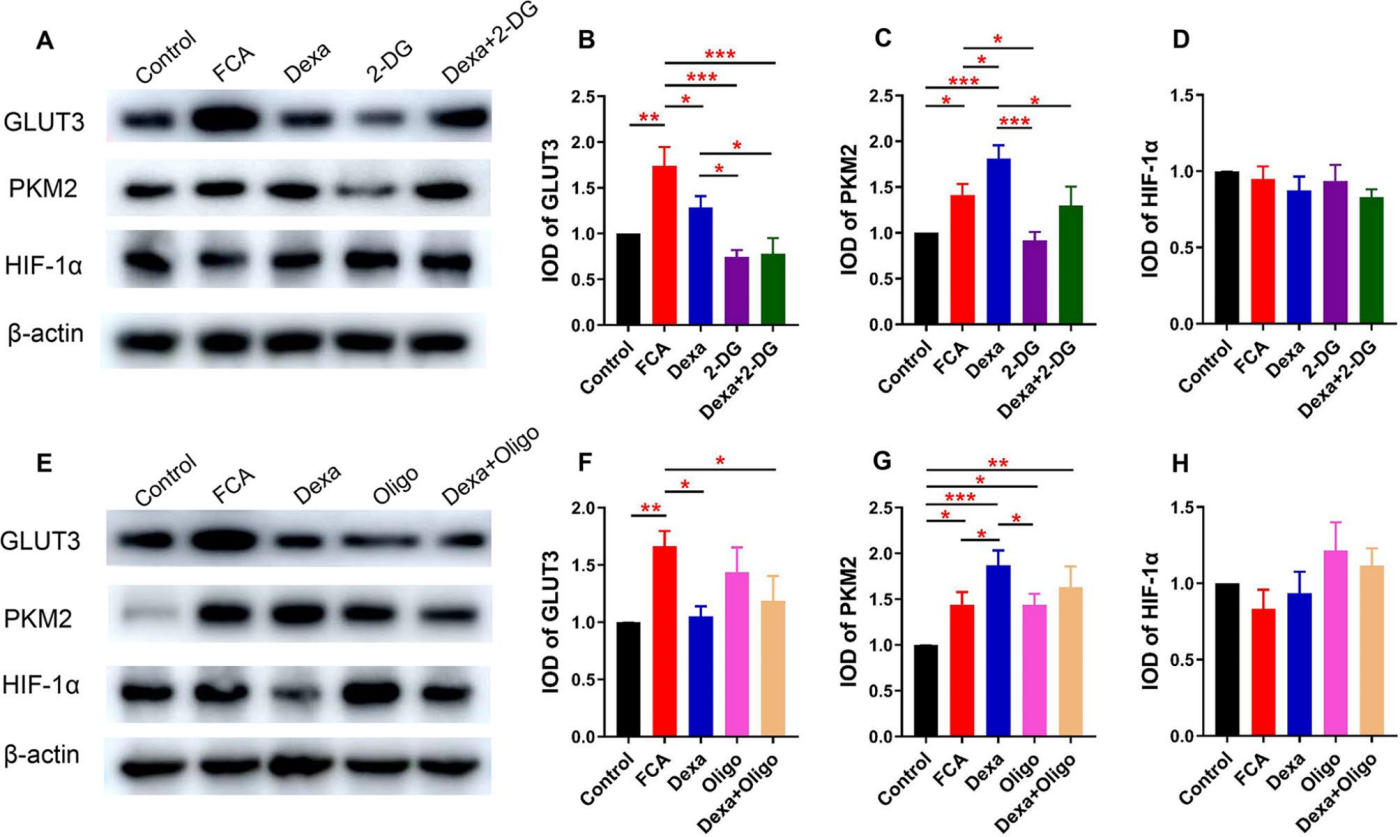
Western blot analysis of the spinal cord proteins in glycolysis and mitochondria-related pathway in inflammatory pain. (**A–H**) The expression of GLUT3, PKM2, HIF-1α in the spinal cord following intrathecal injection of Dexa (20 µg/20 µL), 2-DG (5 mg/20 µL), Oligo (5 µg/20 µL), and the combinations Dexa 20 µg + 2-DG 5 mg/20 µL and Dexa 20 µg + Olig 5 µg/20 µL. Dexa significantly decreased GLUT3 and increased the PKM2 levels in FCA-treated mice, respectively, except HIF-1α. 2-DG reduced the expression of both GLUT3 and PKM2 (but not HIF-1α), whereas Oligo did not change their level during inflammatory pain. Data were normalized against β-actin and are expressed as ratios of control. Data are shown as mean ± SEM (n = 4–6). * *P*<0.05; ** *P*<0.01; and *** *P*<0.001; one-way ANOVA with Fisher-LSD tests. FCA, Freund’s complete adjuvant. GLUT3, glucose transporter isoform 3; PKM2, pyruvate kinase M2; HIF-1α, hypoxia inducible factor-1α

Our results showed that Dexa downregulated GLUT3 and upregulated the PKM2 levels in the FCA-treated mice, but did not influence the HIF-1α expression. 2-DG inhibited GLUT3 and PKM2 expression, whereas Oligo did not have this effect (Fig. 2).

### Dexa inhibited the mitochondrial pathway-related proteins of Mfn2

**Fig. 3:**
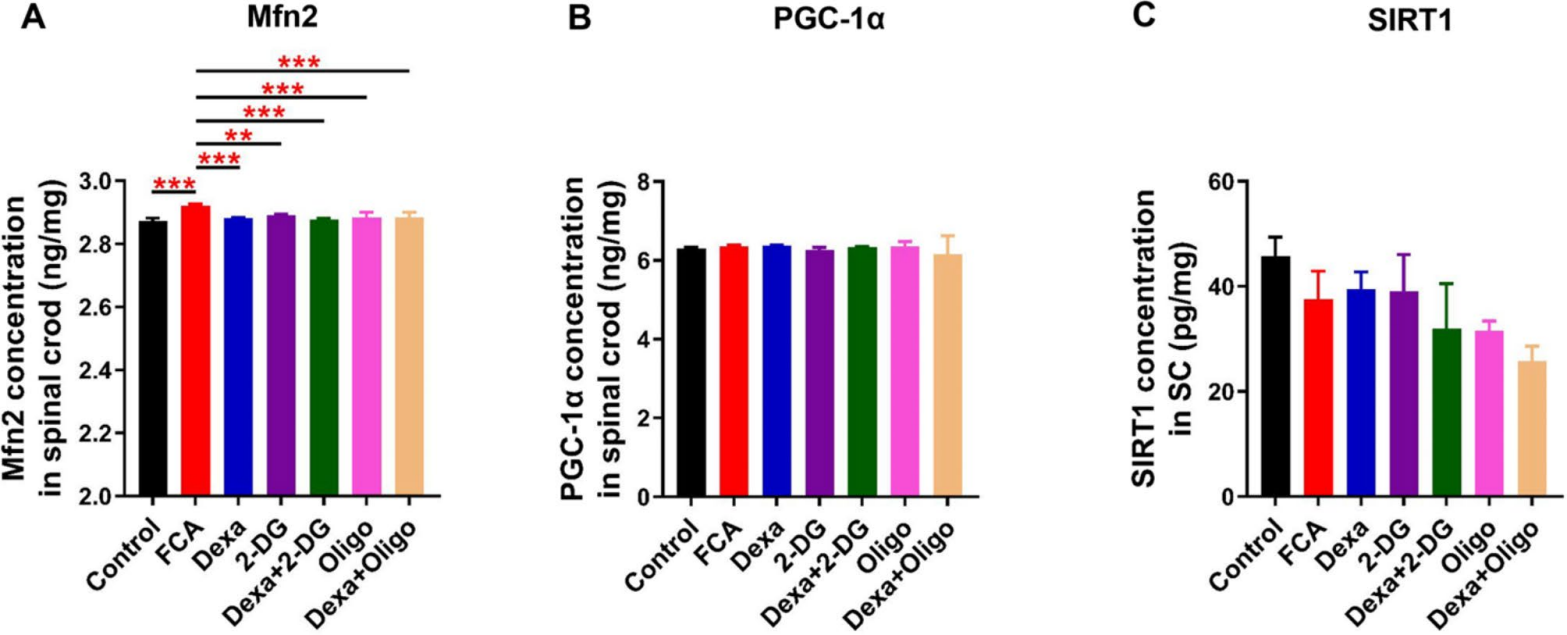
Levels of proteins in glycolysis and mitochondria-related pathway detected by ELISA in the spinal cord. (**A–C**) Both Dexa and 2-DG, Oligo attenuated Mfn2 expression without any change of PGC-1α and SIRT-1 level during FCA-induced inflammation. Data are expressed as mean ± SD (n = 4–6). * *P*<0.05; ** *P*<0.01; and *** *P*<0.001, one-way ANOVA with Bonferroni tests. FCA, Freund’s complete adjuvant; Mfn2, Mitofusin 2; PGC-1α, peroxisome proliferators-activated receptor γ coactivator alpha (PGC-1α); SIRT1, silent mating-type information regulation 2 homolog- 1;

Dexa significantly decreased Mfn2 expression in the spinal cord tissue, while the levels of SIRT-1 and PGC-1α remained unchanged under inflammatory conditions (Fig. 3). Moreover, the administration alone or in combination of Dexa and 2-DG, Oligo also downregulated more significantly the Mfn2 expression as compared to that in the FCA group (Fig. 3A).

### Dexa may modulate PKM2 expression via its activator fructose-1,6-bisphosphate (FBP)

**Fig. 4:**
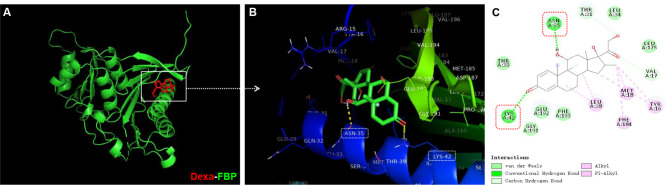
Molecular docking of Dexa and fructose-1,6-bisphosphate (FBP, PKM2 activator). (**A**) Dexa (red) bound to FBP (green) with a binding energy of -7.7 kcal/mol in the docked model; (**B**) Magnified view of the docked structure (in the white box) indicating the interaction between Dexa and FBP; (**C**) Two-dimensional ligand-molecular (Dexa-FBP) interactions diagram. Dexa interacts with the residues of the binding of FBP mainly via the hydrogen bond and van der Waals forces

From the molecular docking results, we can see that Dexa forms two hydrogen bonds with ASN35, LYS42, THR31, and VAL17 of FBP and interacts through van der Waals forces with THR39, LEU34, LEU175, GLY191, GLU192, and PHE193 (Fig. 4).

### Dexa inhibit IL-8 production via glycolytic and mitochondrial pathway

**Fig. 5:**
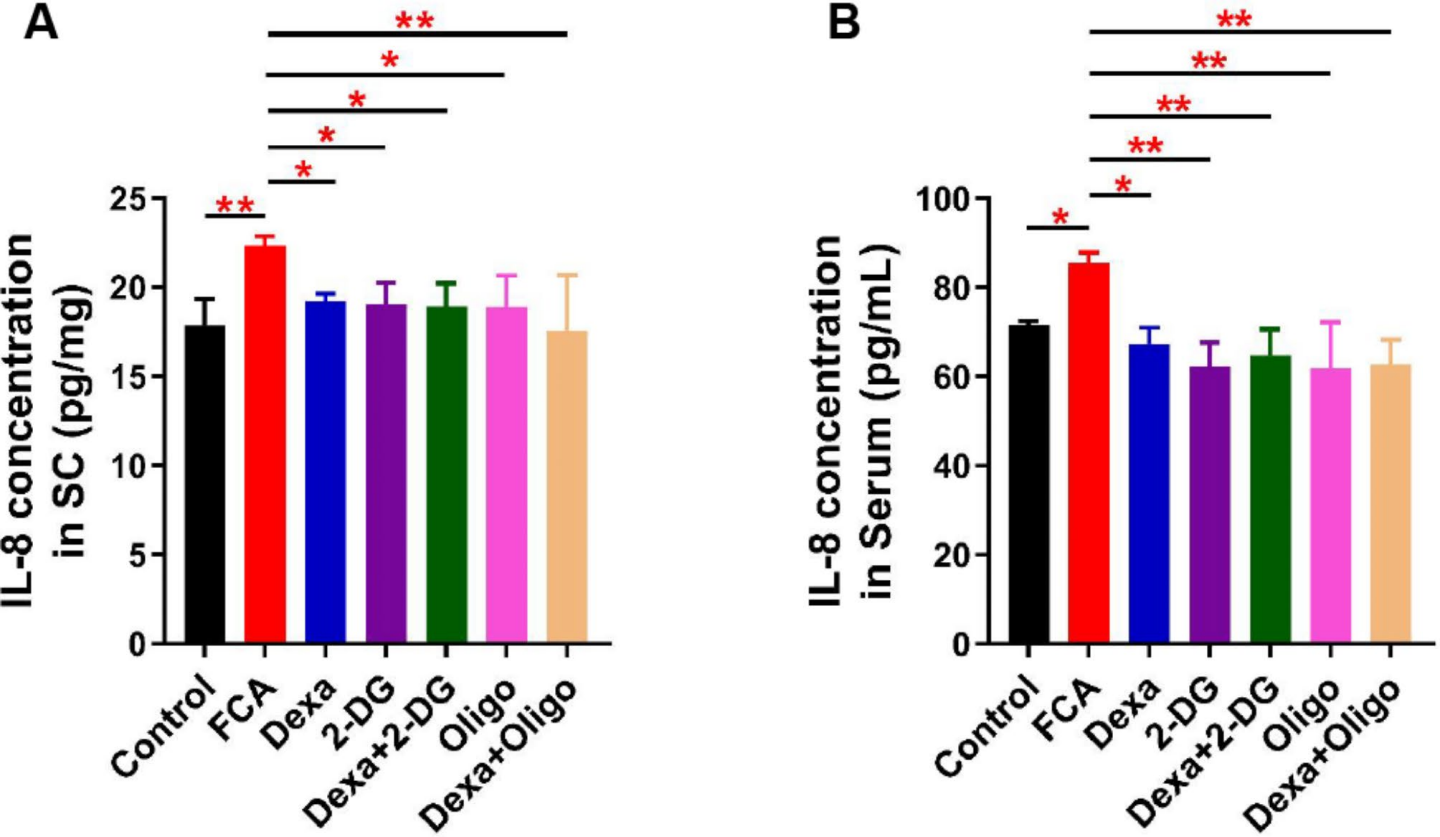
IL-8 concentration levels were tested by ELISA. Intrathecal injection of Dexa and/or 2-DG, Oligo significantly decreased IL-8 expression in both the spinal cord and the serum. Data are shown as mean ± SD (n = 4–6). * *P*<0.05; ** *P*<0.01; and *** *P*<0.001, one-way ANOVA with Fisher-LSD tests

As can be seen from the results, the level of IL-8 significantly decreased in both the spinal cord tissue and the serum following the administration of Dexa alone or the that of the combination of Dexa and 2-DG, Oligo (Fig. 4 and 5), suggesting that IL-8 is a downstream protein of the Dexa metabolism pathway (glycolytic and mitochondrial pathways) and is involved in the regulation of inflammatory pain sensation.

## Discussion

**Fig. 6:**
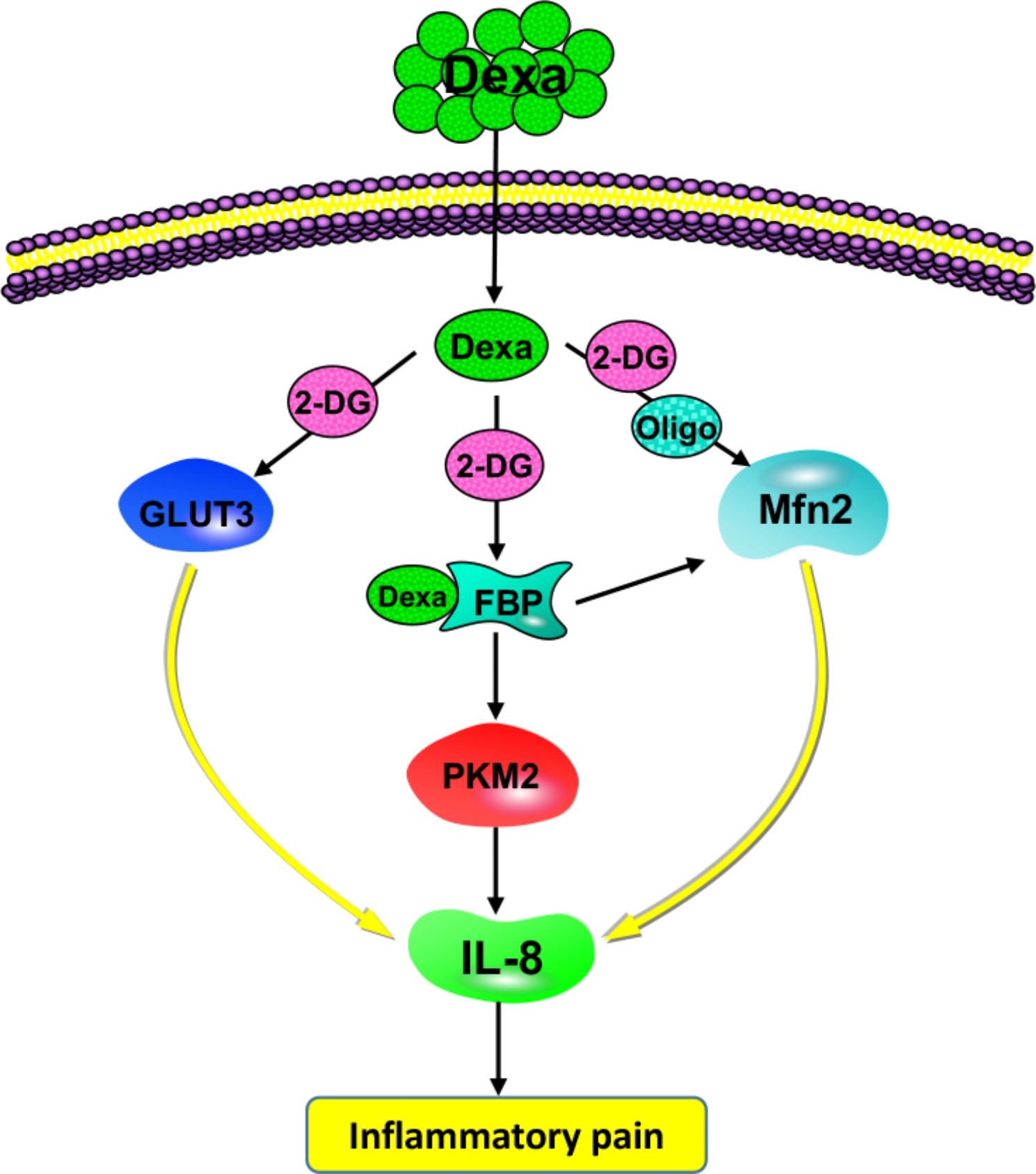
Dexa reduced IL-8 expression via glycolysis and mitochondrial pathway-related proteins, thus alleviating pain under inflammatory conditions. Intrathecal injection of Dexamethasone (Dexa) decreased the levels of GLUT3 (glycolysis-related protein) and Mfn2 (mitochondrial pathway-related protein), and further increased the PKM2 (glycolysis-related protein) expression. The antinociceptive effects of Dexa were counteracted by 2-DG (glycolysis inhibitor) or Oligo (mitochondrial pathway inhibitor) application. The upregulation of PKM2 induced by Dexa administration might be due to the stable Dexa-PKM2 binding, thus reducing the PKM2 activity and the quantity of the remaining PKM2.

As can be seen in Fig. 6, our study revealed that the analgesic action of Dexa inhibited IL-8 during inflammatory pain through the glycolysis and mitochondria-related pathways, indicating that metabolism pathway and IL-8 contributed to the occurrence of inflammatory nociception.

In the present study, we adopted local intraplantar administration of FCA to induce inflammatory pain model in mice, leading to a significant decrease in PWMT. As expected, intrathecal injection of Dexa inhibited this pain response and showed powerful antinociceptive action within 60 min (Fig. 1), which is in agreement with the findings in previous literature reports [1, 16, 17]. Interestingly, this analgesic effect of Dexa was reversed by 2-DG (glycolysis inhibitor) or Oligo (mitochondrial pathway inhibitor) application (Fig. 1). These results revealed that Dexa exerted analgesic effect via the glycolysis and mitochondria-related pathways. To the best of our knowledge, there have been no previous reports indicating that 2-DG or Oligo is effective in counterbalancing the antinociceptive action of Dexa. Therefore, our present results extend the existing knowledge of the analgesic mechanism of Dexa.

After we found that both 2-DG and Oligo can counteract Dexa-induced analgesic effect, we detected the glycolysis and mitochondrial pathway-related proteins. As we know, the upregulation of GLUT3 promotes intracellular glucose breakdown though glycolysis [18]. Here, we found that Dexa decreased GLUT3 expression (Fig. 2), which contributed to a reduced glucose entry, interfering with glycolysis. Curiously, the increase in PKM2 was observed after the administration of Dexa during FCA-induced inflammation (Fig. 2). The most likely reasons for this phenomenon are as follows: (1) Molecular docking results indicated that Dexa formed hydrogen bonds with FBP (Fig. 4), and this stable compound limited the activational effects of FBP. The presence of FBP was documented to significantly decrease the promotion of proliferation induced by Dexa [19], which might have attributed to complex formation; (2) Dexa was experimentally confirmed to reduce PKM2 activity [20], and the low catalytic activity of PKM2 inhibited the glycolytic pathway in the final step, leading to intracellular accumulation of intermediate products and remaining enzymes (e.g., PKM2) within the cells [21].

Our results showed that Dexa decreased Mfn2 expression in FCA-treated mice (Fig. 3). As it has been reported in the literature, the level of Mfn2 in the spinal cord was suppressed by Dexa administration [22], which is consistent with the present findings. As illustrated in Figs. 2 and 3, GLUT3 and PKM2 were inhibited by 2-DG (but not Oligo), and Mfn2 was suppressed by both 2-DG and Oligo. These effects could have occurred because Mfn2 is an upstream factor and it could affect glycolysis through decreasing PKM2 activity and binding to PKM2 [23]. This is somewhat contrary to the results published in earlier literature reports where the overexpression of PKM2 was found to increase the Mfn2 expression [24], suggesting that both glycolytic and mitochondria-related proteins were able to influence each other.

Furthermore, no differences of HIF-1α, PGC-1α, and SIRT1 expression were also observed in this study, suggesting that these factors likely played an insignificant role in the spinal cord during inflammatory pain. Finally, Dexa was confirmed to reduce IL-8 levels in the spinal cord and the serum (Fig. 5), which is in line with previously reported results [15, 25]. There is currently not evidence in the literature that a similar reduction in IL-8 expression has been observed after the administration of an intrathecal injection of 2-DG or Oligo, revealing that both the glycolysis and mitochondrial pathways participated in the modulation of IL-8 during inflammatory processes.

Although our research provides novel evidence, some limitations require attention. First, the molecular docking was employed for the mimicking of Dexa and FBP to explain the phenomenon of the increased expression of PKM2 by Dexa application, but we did not validate the in vitro interaction between Dexa-FBP and PKM2 activity. Second, we did not investigate a large number of molecules involved in the glycolysis- (e.g., NRF2 and NLRP3) [26, 27] and mitochondria-related (e.g., mtDNA and SMAC) [28, 29] pathways. Finally, further detection and analysis of cytokine levels could not be done in our experiment. Previous results indicated that pro-inflammatory factors (e.g., IL-1β, IL-6, and IL-17) and anti-inflammatory cytokines (e.g., IL-4, IL-10, and IL-18) might exert important effects during inflammation and pain [30, 31].

In conclusion, our results reveal that Dexa is capable to attenuate the increase in the IL-8 level via glycolysis-related (GLUT3 and PKM2) and mitochondrial-related (Mfn2) pathway proteins, thereby regulating the analgesic effect in inflammatory pain.

### Electronic supplementary material

Below is the link to the electronic supplementary material.

Supplementary Material 1

Supplementary Material 2

Supplementary Material 3

Supplementary Material 4

Supplementary Material 5

Supplementary Material 6

## Data Availability

The datasets used and/or analyzed during the current study are available from the corresponding author on reasonable request.

## Abbreviations

Dexa: dexamethasone
GLUT3: glucose transporter isoform 3
PKM2: pyruvate kinase M2
HIF-1α: hypoxia inducible factor-1α
Mfn2: mitofusin 2
PGC-1α: peroxisome proliferators-activated receptor γ coactivator alpha
SIRT1: silent mating-type information regulation 2 homolog-1
IL-8: interleukin 8
FCA: freund’s complete adjuvant
2-DG: 2-deoxyglucose
Oligo: oligomycin complex
PWMT: paw withdrawal mechanical threshold
ELISA: enzyme-linked immunosorbent assay
FBP: fructose-1,6-bisphosphate
