# Supplementary figures and images for: Dexamethasone inhibits IL-8 via glycolysis and mitochondria-related pathway to regulate inflammatory pain
Source: BMC Anesthesiol. 2023 Sep 18;23:317. doi: 10.1186/s12871-023-02277-9 (PMC10506226; doi:10.1186/s12871-023-02277-9)

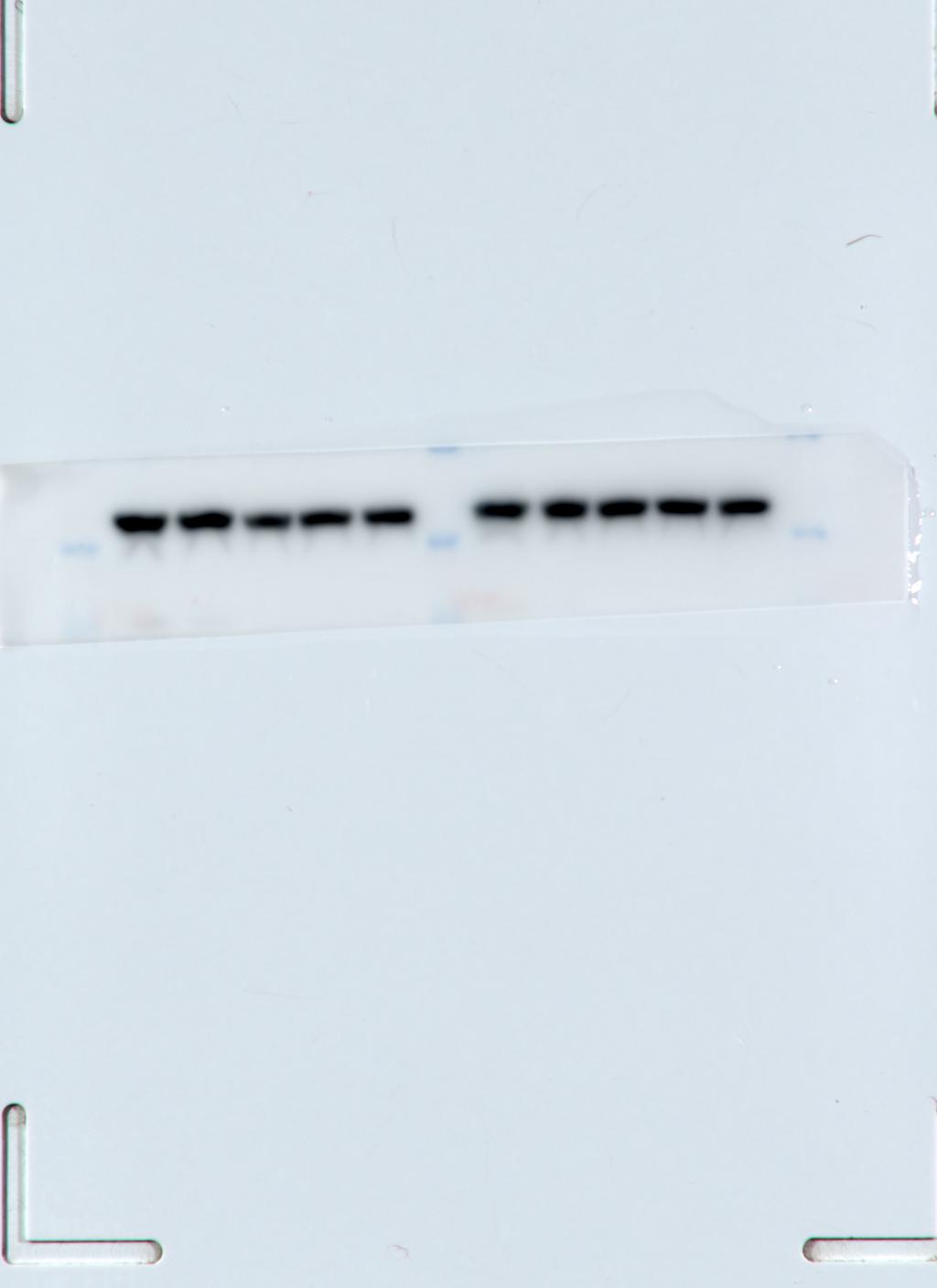

Supplement: Supplementary file 1 — Supplementary Material 1 [file 12871_2023_2277_MOESM1_ESM.jpg]

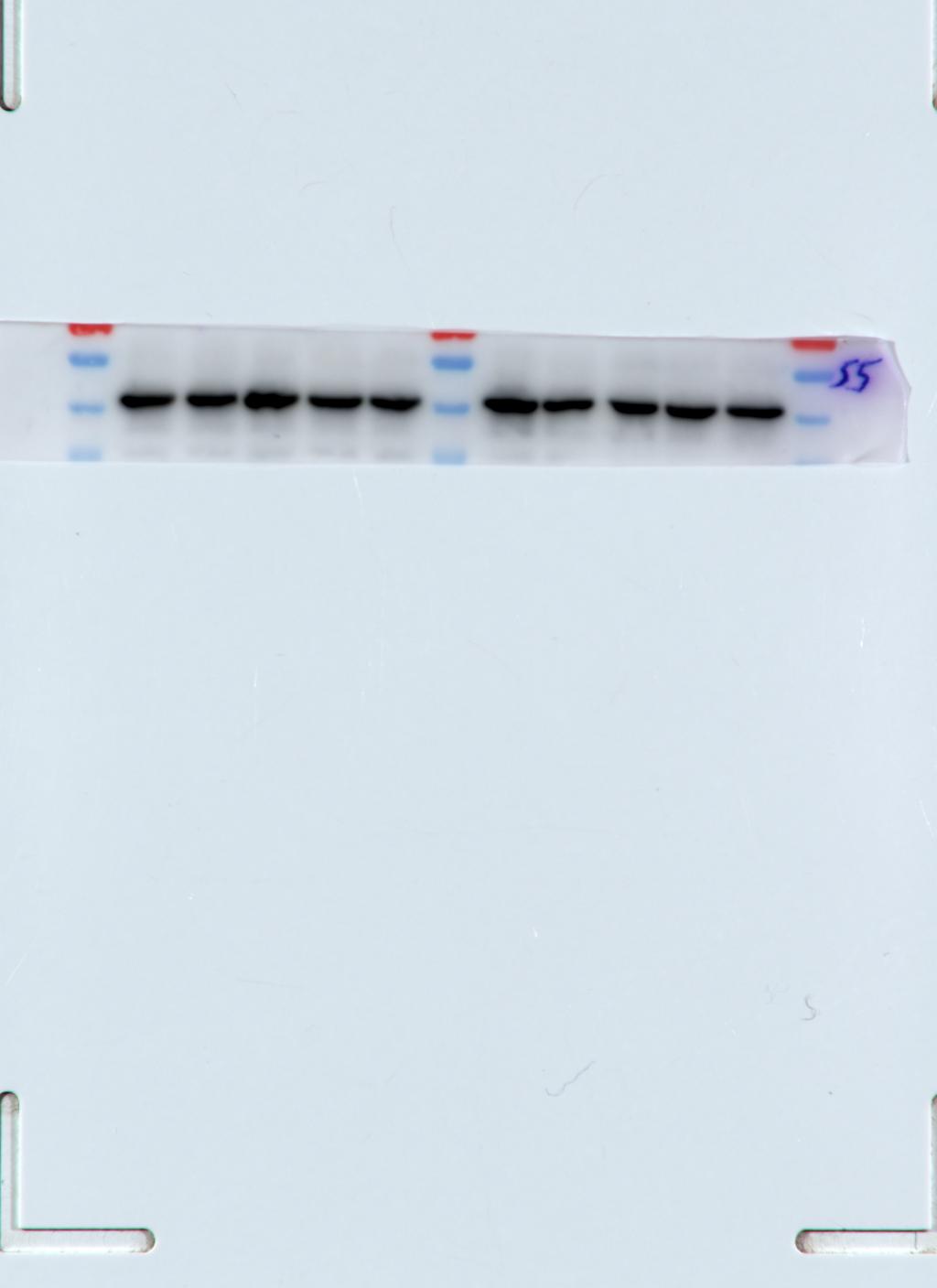

Supplement: Supplementary file 2 — Supplementary Material 2 [file 12871_2023_2277_MOESM2_ESM.jpg]

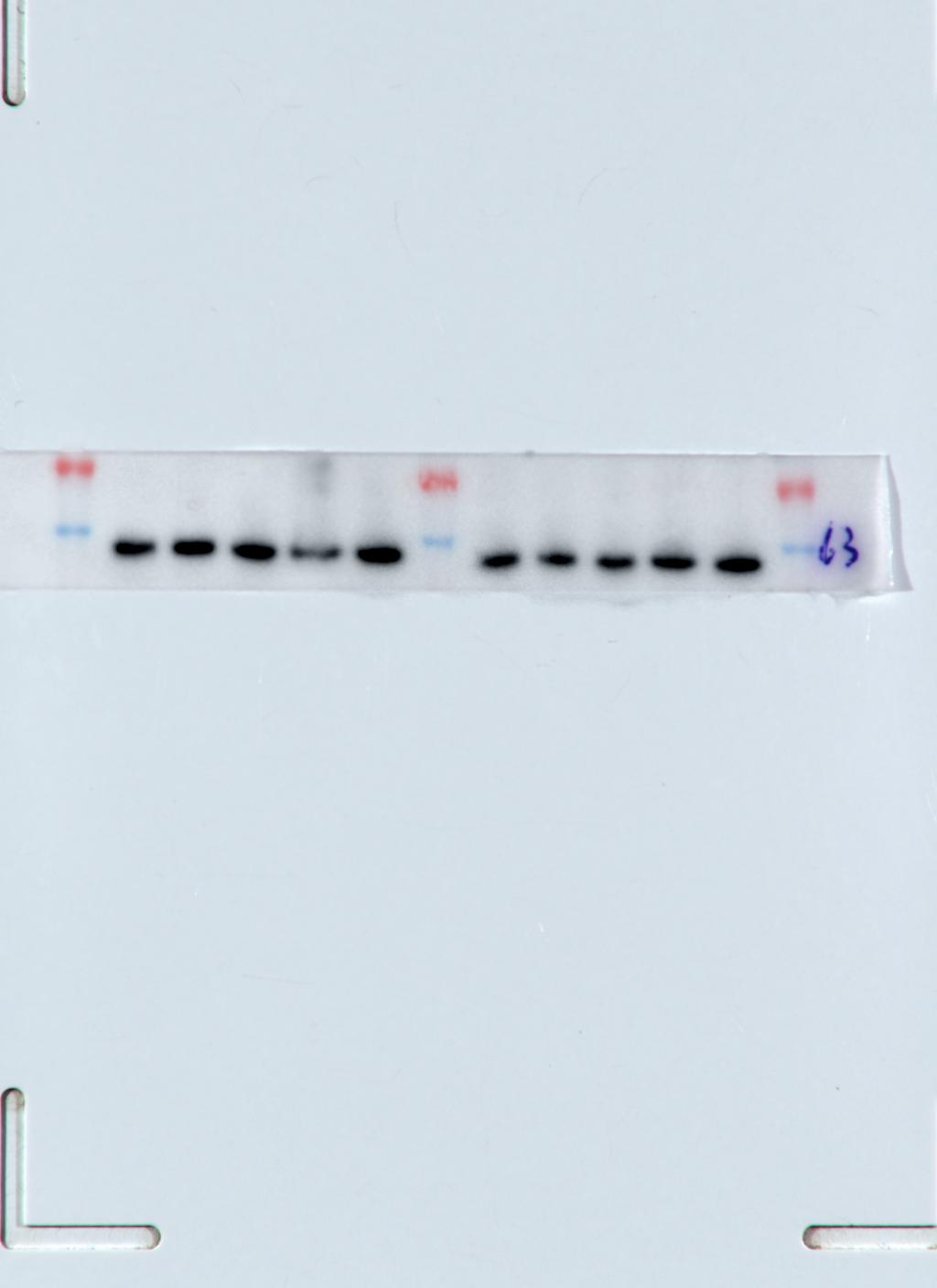

Supplement: Supplementary file 3 — Supplementary Material 3 [file 12871_2023_2277_MOESM3_ESM.jpg]

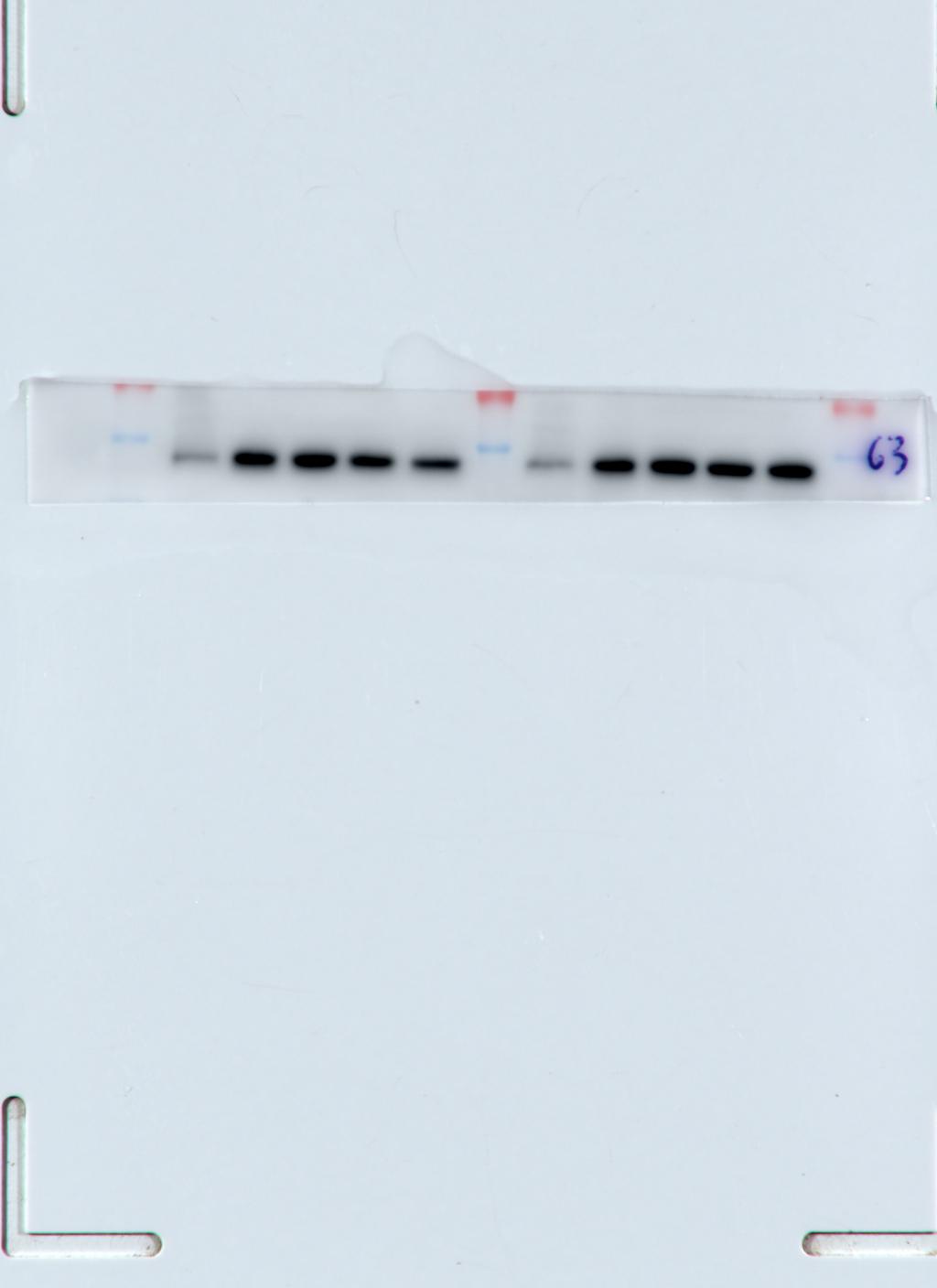

Supplement: Supplementary file 4 — Supplementary Material 4 [file 12871_2023_2277_MOESM4_ESM.jpg]

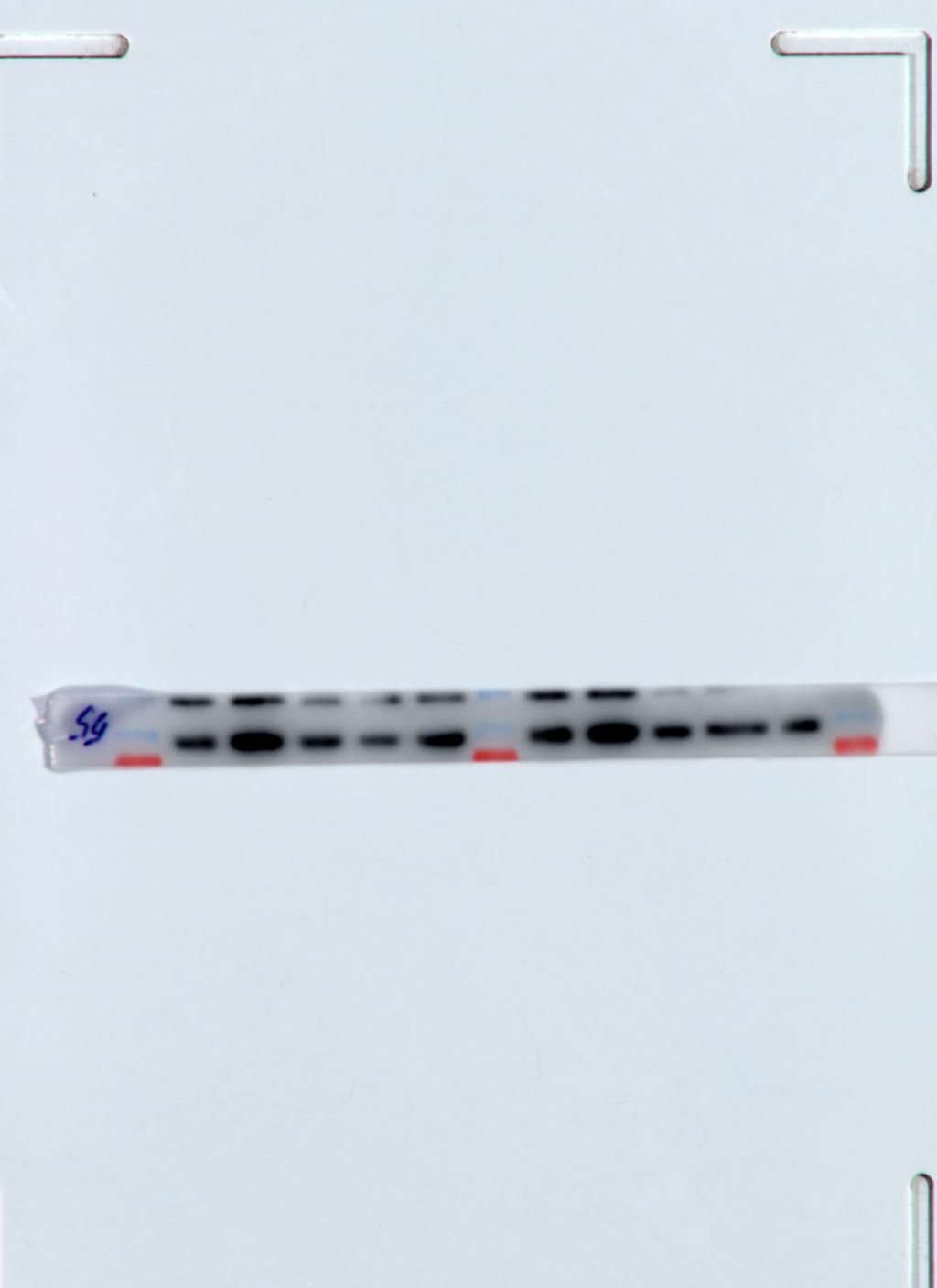

Supplement: Supplementary file 5 — Supplementary Material 5 [file 12871_2023_2277_MOESM5_ESM.jpg]

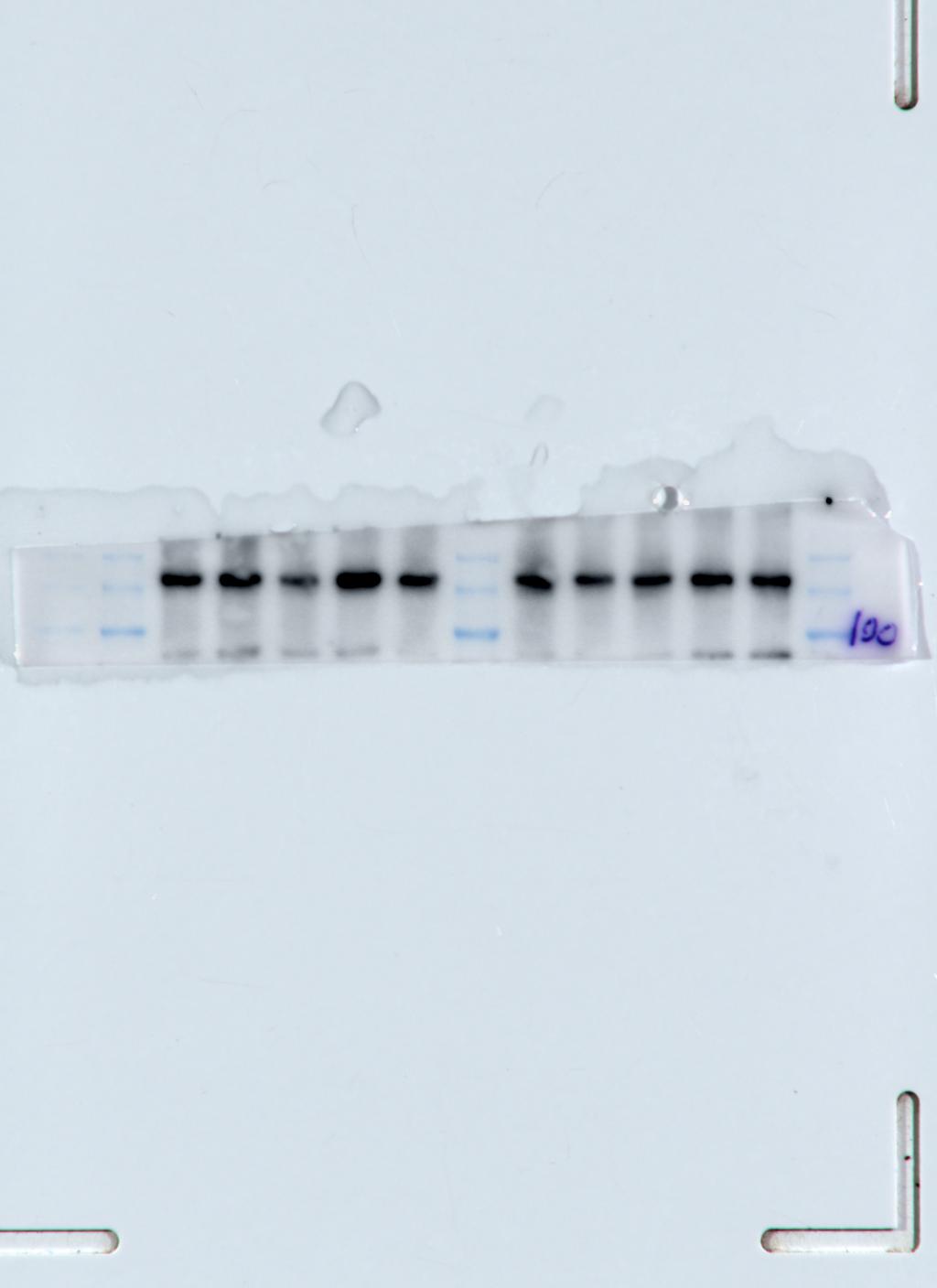

Supplement: Supplementary file 6 — Supplementary Material 6 [file 12871_2023_2277_MOESM6_ESM.jpg]
